# Propolis as an alternative treatment for companion animal skin infections: addressing antimicrobial resistance

**DOI:** 10.3389/fvets.2026.1880076

**Published:** 2026-10-22

**Authors:** Cherrihan Adra, Malin A. Olsson, Remona Horn, Trong D. Tran

**Affiliations:** 1 Centre for Bioinnovation, University of the Sunshine Coast, Sippy Downs, QLD, Australia; 2 School of Science, Technology and Engineering, University of the Sunshine Coast, Sippy Downs, QLD, Australia; 3 Eumundi Vets, Eumundi, QLD, Australia

**Keywords:** antimicrobial resistance, bee products, companion animals, One Health, propolis, skin infections, veterinary dermatology

## Abstract

Bacterial skin infections are among the most prevalent health concerns in companion animals and represent a primary driver for antimicrobial prescriptions within veterinary practices. The widespread use of empirical broad-spectrum antibiotic prescribing has contributed to the emergence of antimicrobial-resistant pathogens, including *Staphylococcus pseudintermedius* and *Pseudomonas aeruginosa* in veterinary dermatology. This resistance complicates treatment outcomes and raises broader public health concerns under the One Health framework. Consequently, there is a growing need for alternative antimicrobial strategies that can reduce the reliance on conventional antibiotics. Propolis, a resinous substance produced by bees, has gained attention as a potential candidate due to its wide range of biological activities including antimicrobial, anti-inflammatory and wound healing. This structured narrative review evaluates the current evidence for propolis in the treatment of dermatologic infections in companion animals. The available evidence demonstrates that propolis exhibits antimicrobial activity against key veterinary dermatologic pathogens including *Staphylococcus* spp., *Pseudomonas aeruginosa*, *Malassezia pachydermatis* and dermatophytes. However, this evidence is primarily based on *in vitro* studies with very limited clinical data. While clinical studies have examined propolis in other veterinary dermatologic applications, no clinical trials to date have specifically investigated its use in the treatment of canine pyoderma, a common and frequently recurring skin infection in canine practice. Recommendations for future research include controlled clinical trials evaluating propolis for the treatment of companion animal skin infections as an adjunct to, or potential alternative for, conventional antibiotics, the development of standardization protocols due to the chemically heterogeneous nature of propolis and refinement of delivery systems. Overall, these findings support the promising potential of propolis as a treatment for skin infections in companion animals.

## 1 Introduction

Skin infections are among the most common ailments in companion animal medicine (1, 2). The management of skin infections in companion animals often requires the use of systemic antimicrobials (3–5) with many studies showing a high proportion of veterinary consultations involve prescription of antibiotics, with a notable preference for broad-spectrum agents (6). For instance, a study surveyed 52 veterinary practices and collated 2,599 records from dogs and cats and found that 29.2% of animals were prescribed antibiotics and 24.6% prescribed systemic antibiotics (7). Another study from the UK analyzed 281,543 consultations from dogs and 111,139 from cats, finding that systemic antimicrobial drugs were prescribed during 25.7% of consultations for dogs and 32.9% for cats (8). Furthermore, many veterinary practices were shown to be conducting empirical prescribing which involves the initiation of treatment without confirmation of culture or sensitivity testing (3, 9). This approach may provide initial relief for the companion animal but carries the risk of selecting for resistant bacterial strains and exacerbating antimicrobial resistance (10, 11). For instance, a study conducted by Cousto et al. (12) looked at the prescription patterns of the antibiotic meropenem over a 7-year period and found that 65.4% of prescriptions were initiated without or prior to culture results. Additionally, retrospective analysis of culture and susceptibility results showed only 2.9% of empirical meropenem selections were required (12). Another study in 2011 analyzed 18,905 cats and dogs referred to the veterinary hospital from 2000 to 2007 and found that less than 5% of antibiotics prescribed had support from microbiological analyses and susceptibility testing (13). The indiscriminate and increasing incidences of antimicrobial-resistant (AMR) infections in veterinary dermatology presents a growing concern as it leads to recurrence and prolonged morbidity for the affected animal (5, 14, 15). Furthermore, it is important to consider the broader implications of inappropriate antimicrobial use in animals in terms of the One Health approach, which underscores the interconnection between animal, human and environmental health. Companion animals can serve as reservoirs and vectors for resistant pathogens with zoonotic bacteria including *Staphylococcus pseudintermedius* (*S. pseudintermedius*) and *Escherichia coli* (*E. coli*) posing potential risks to human health via direct contact or environmental contamination (16, 17). Therefore, the burden of skin infections in animals is not an isolated clinical issue but rather a significant component within the broader framework of AMR mitigation. Given these challenges, there is growing interest in exploring alternative antimicrobial therapies that can reduce reliance on conventional antibiotics and mitigate the development of resistance.

Propolis, commonly known as bee glue, is a natural substance produced by bees and offers a promising alternative when it comes to treating skin infections (18–22). It is made up of plant resins (50%), beeswax (30%), essential oils (10%), pollen (5%), and other organic substances including bee salivary enzymes (5%) (23, 24). Bees use this mixture to construct, seal, repair and protect their hives from invading microorganisms (25–27). Over 500 chemical compounds have been identified from propolis including polyphenols, fatty acids, terpenoids, flavonoids, steroids, aromatic alcohols and aldehydes (28). However, its chemical composition is very much influenced by the region and plant sources from which bees are foraging (23, 29). Propolis has been used for centuries in traditional medicine and has been reported to exhibit various biological activities including antibacterial, antifungal, antioxidative, anti-inflammatory and anticancer activities (30, 31). Importantly, propolis exerts its antimicrobial effects through multiple mechanisms simultaneously which collectively may reduce the likelihood of resistance development, although this has not been confirmed in longitudinal resistance studies (32–35). It has also demonstrated its efficacy against both Gram-positive and Gram-negative veterinary dermatologic pathogens (20, 36–39). While previous reviews have examined the broader therapeutic use of propolis in veterinary medicine (18, 40), none have specifically evaluated the evidence of propolis in the treatment of dermatologic infections in companion animals.

## 2 Search strategy and selection criteria

This review was conducted as a structured narrative review. While the heterogeneity of the available literature precludes a formal systematic review with meta-analysis, a standardized search and screening protocol was adopted. A literature search was conducted using three electronic databases: PubMed, Scopus, Web of Science, with the following search string: TITLE-ABS-KEY(propolis) AND TITLE-ABS-KEY(dog OR dogs OR canine OR cat OR cats OR feline OR “companion animal*”) AND TITLE-ABS-KEY(skin OR dermatology OR infection OR pyoderma OR otitis OR wound). Only peer-reviewed articles published focusing on bacterial and fungal dermatologic conditions in companion animals were included. Studies were included if they met the following criteria: (i) evaluated propolis in any form or preparation for antimicrobial activity; (ii) targeted bacterial, fungal or mixed dermatologic conditions of the skin, ear or associated soft tissues; (iii) involved companion animal species (dogs, cats) or used pathogens isolated from companion animal clinical cases; and (iv) were published in English. Records retrieved from database searches were imported into Rayyan^1^ (41) for dereplication and screening (Figure 1).

**Figure 1:**
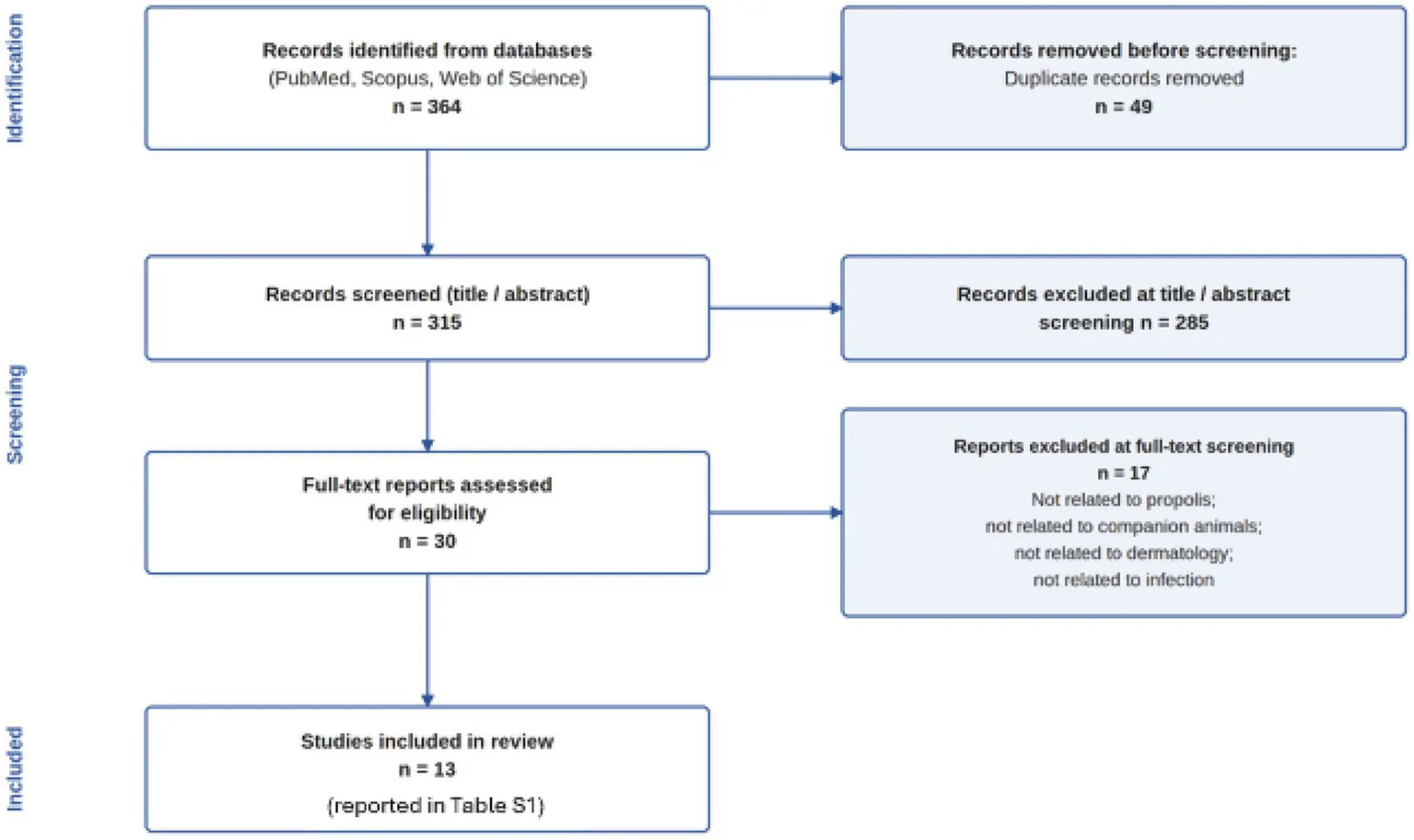
PRISMA flow diagram of study identification, screening and inclusion.

## 3 Clinical burden and AMR implications of dermatologic infections in companion animals

Common bacterial skin infections in companion animals include superficial or deep pyoderma, otitis, bite wounds and abscesses (42). The primary causative agents associated with these conditions include *S. pseudintermedius*, *Pseudomonas aeruginosa* (*P. aeruginosa*), *E. coli*, *Proteus mirabilis* (*P. mirabilis*), *Enterococcus* spp., and *Malassezia* spp., many of which have demonstrated antimicrobial resistance (2, 42–44). The following subsections describe the clinical burden, microbiology and current treatment strategies associated with each condition.

### 3.1 Pyoderma

Pyoderma is one of the most common dermatologic conditions in dogs, characterized as a purulent bacterial skin infection that significantly impacts the health and welfare of the affected animal (45, 46). This condition arises from disruptions to local skin defense mechanisms, leading to secondary bacterial invasion (46) and can be categorized into three main types, consisting of surface, superficial and deep. Superficial pyoderma, the most frequently encountered form, refers to the epidermis, extending into the upper portion of the hair follicle (47, 48). This occurs when bacteria proliferate on the surface of the skin and incite an inflammatory response without invading the deeper tissue of the skin (46). The predominant bacterial pathogen responsible for this skin infection is *S. pseudintermedius*, with 92% of canine pyoderma cases reported to be caused by this pathogen (49). Other pyoderma associated species include *S. delphini*, *S. schleiferi*, and *S. aureus*, the latter being more commonly implicated with feline pyoderma (4, 47). The dominance of *S. pseudintermedius* is well supported with one study isolating 65 bacterial strains from dogs and found 72.3% belonged to *S. pseudintermedius* and 18.5% to other *Staphylococcus* spp. (50). Of particular concern is the emergence of multidrug-resistant *S. pseudintermedius*, which acquires resistance through mobile genetic elements such as *Staphylococcal* cassette chromosome *mec* (SCC*mec*). The *mec*A gene enables bacteria to produce a penicillin-binding protein (PBP2a) with reduced affinity for β-lactam antibiotics, conferring methicillin resistance (50). This resistance has been reported in up to 60% of *S. pseudintermedius* strains isolated from canine pyoderma (51, 52). A 2022 study assessed 114 *Staphylococcus* isolates (*S. aureus* from 43 cats and 19 dogs, and *S. pseudintermedius* from 11 cats and 41 dogs) for sensitivity against 24 antimicrobial agents. The most prevalent form of resistance was against penicillin observed via the *BlaZ* gene (76–90%), followed by macrolide resistance (27%) via *erm* genes (encoding ribosomal RNA methylases that prevent macrolide, lincosaminde and streptogramin B binding), methicillin resistance (25%) via *mec*A or *mec*C, fluoroquinolone resistance (21%) and tetracycline resistance (19.3%) via *tet* (K) (encoding an efflux pump) and/or *tet* (M) (encoding a ribosomal protection protein) (53). This resistance profile poses a serious issue given the antibiotic classes routinely used for systemic treatment of pyoderma include β-lactams, lincosamides, sulfonamides, tetracyclines, fluoroquinolones, aminoglycoside, phenicols, and rifamycins (54). Commonly prescribed antibiotics for pyoderma infections include amoxicillin-clavulanic acid (penicillin/β-lactams), cephalexin (cephalosporin), clindamycin (lincomycin) and cefovecin (cephalosporin) (44). Current prescribing practices for pyoderma may be contributing to this resistance selection. One study investigated 54,600 dogs presented to 73 practices and despite only 1.3% having recorded a pyoderma diagnosis, 97% of the cases were prescribed antimicrobials with 92% receiving systemic therapy (55). Similarly, another study identified 683 canine pyoderma cases and found that 64.1% were prescribed systemic antimicrobials and 27.7% received both systemic and topical therapies concurrently (56). The combination of high empirical prescribing rates and the documented emergence of resistant genes in pyoderma-associated pathogens underscores the need for alternative treatment strategies.

### 3.2 Otitis

Otitis externa is the inflammation of the external ear canal from the pinna to the tympanic membrane and is a common diagnosis, accounting for 15–20% of canine disorders (57, 58). Clinical signs include inflammation, pain, discharge, excessive scratching and head shaking (Figure 2B) (59). Among bacterial pathogens, *P. aeruginosa*, a Gram-negative opportunistic bacterium, is the most frequently isolated reported in 18–35% of canine otitis cultures (60). Other causative agents of otitis infection include *S. pseudintermedius*, *E. coli*, *Klebsiella* spp. and the yeast *Malassezia* spp. (61, 62). One study investigated results obtained from a veterinary diagnostics laboratory from 2020–2022 with a total of 604 cases of canine external otitis and found the most prevalent microorganisms were *P. aeruginosa* (30.7%), *P. mirabilis* (29.4%), *S. pseudintermedius* (25.1%) and *E. coli* (19%), with 47% of isolates presenting with multidrug-resistant phenotypes (63). Treatment is particularly challenging when it comes to *P. aeruginosa* due to its intrinsic resistance mechanisms, capacity to rapidly acquire additional resistance and its ability to form biofilms which was found in 40–95% of *P. aeruginosa* cases from otitis externa (64, 65). Biofilms protects bacterial communities from both host immune responses and antimicrobial penetration, often resulting in therapeutic failures and chronic or recurrent infections (66), with reported recovery rates of no more than 35% of otitis media cases (64). Resistance in *P. aeruginosa* is largely gene-mediated, involving chromosomally encoded AmpC β-lactamase, upregulated efflux systems (MexAB–OprM and MexXY) and loss of the OprD porin, which together reduce the activity of β-lactams, aminoglycosides and fluoroquinolones (67). Current treatment relies primarily on topical antibiotics including aminoglycosides such as gentamicin and polymyxin-B, and fluoroquinolones such as marbofloxacin, orbifloxacin, and enrofloxacin (65, 66). However, the high prevalence of multidrug-resistant isolates documented in otitis cases highlight the limitations of current therapeutic approaches and the need for alternative or adjunct treatments.

**Figure 2:**
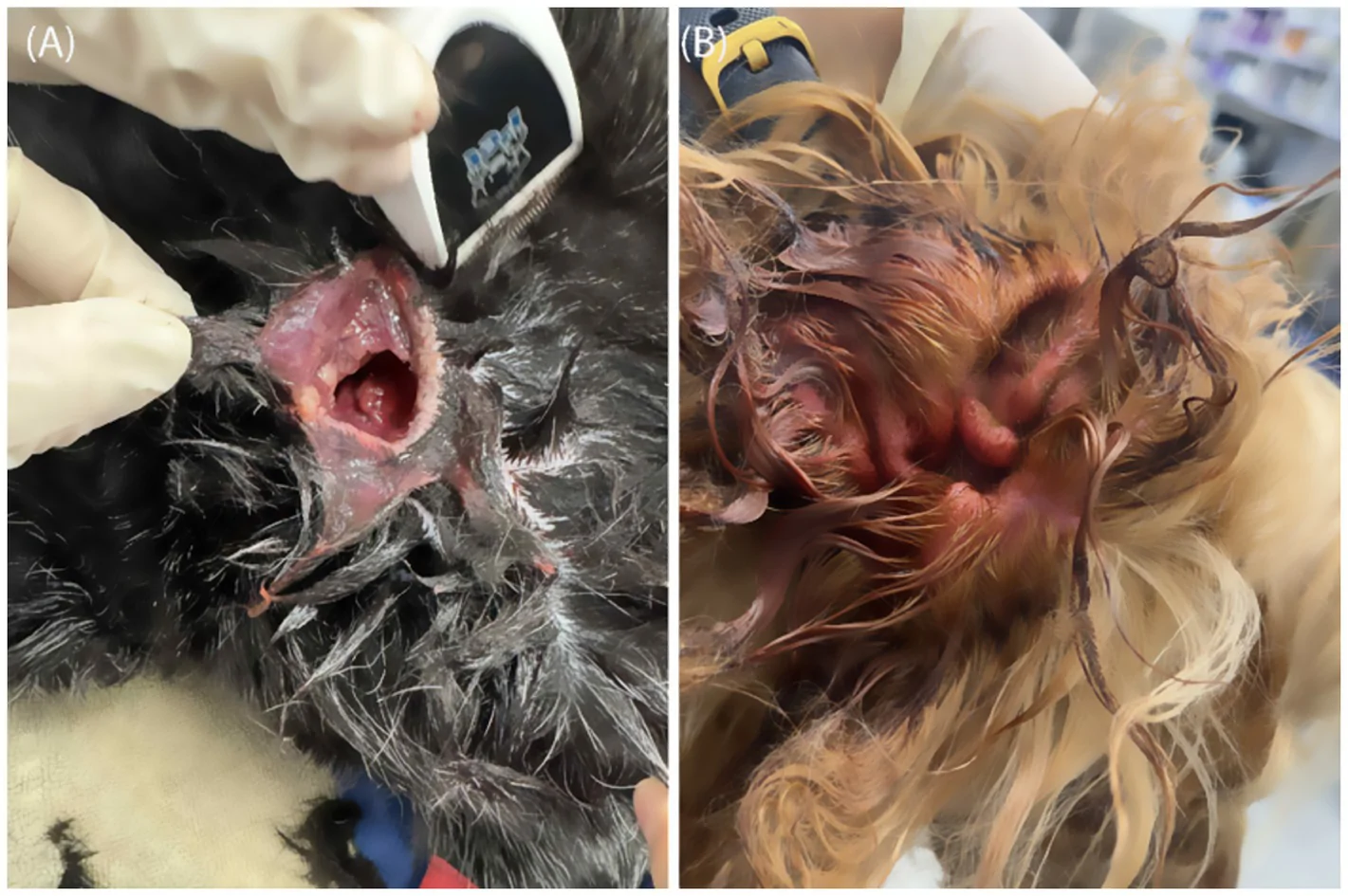
Skin infections in canine patients. **(A)** Infected wound from dog bite and **(B)** Otitis externa (images courtesy of Eumundi Vets veterinary clinic).

### 3.3 Bite wounds

Bite wounds are common traumatic injuries in companion animals, with cats having a slightly higher incidence, accounting for approximately 10–20% of traumatic injuries (68, 69). These injuries facilitate the introduction of pathogenic organisms into deeper tissues, resulting in localized inflammation, swelling, purulent discharge and tissue necrosis (Figure 2A) (69). Bite wound infections are typically polymicrobial, derived primarily from the oral flora of the biting animal and occasionally from the victim’s skin flora or the environment (70, 71). Bacterial flora associated with bite wounds are linked with diverse aerobic and anaerobic organisms including *Pasteurella*, *Staphylococcus*, *Streptococcus*, *Pseudomonas*, *Neisseria*, *Corynebacterium*, *Moraxella* and *Capnocytophaga* species among the most common aerobic and *Fusobacterium*, *Bacteroides*, *Porphyromonas* and *Prevotella*, C*utibacterium* anaerobic (70, 72, 73). *Pasteurella* spp. is the most frequently implicated organism in both dog and cat bites and can develop rapidly often causing infections within 24 h of the bite taking place (74, 75). Acquired resistance is less well characterized among the fastidious organisms of bite-wound infections; nonetheless, β-lactamase production has been reported in *Pasteurella* and *Bacteroides* spp., underlying the reliance on β-lactamase-inhibitor combinations (70). Management of bite wounds often involves empirical broad-spectrum antibiotic therapy to cover both aerobic and anaerobic organisms, particularly in cases of deep puncture wounds, significant tissue damage or when there is a general high risk of infection where contamination is suspected. Amoxicillin-clavulanate is considered the first-line prophylactic antibiotics for bite wounds due to its broad-spectrum efficacy, covering pathogens such as *Staphylococcus*, *Streptococcus* and *Pasteurella*, with cefovecin (third-generation cephalosporin), clindamycin and fluoroquinolone used as alternatives (76).

### 3.4 Abscesses

Abscesses are localized accumulations of purulent exudate beneath the surface of the skin that commonly arise due to penetrating injuries such as bite wounds or punctures, when the overlaying epidermis seals and encapsulates the infected wound (47). Abscesses are usually a result of opportunistic flora from a portal of entry, often presenting as polymicrobial cultures of both aerobes and anaerobes (77). Commonly isolated aerobes include *Pasteurella* spp., *Staphylococcus* spp., *Streptococcus* spp. and *E. coli*, while *Bacteroides*, *Fusobacterium* and *Actinomyces* predominate among anaerobic isolates (78, 79). Management of abscesses centers around prompt surgical drainage and flushing, followed by systemic antimicrobial therapy. Extended courses of broad-spectrum, systemic antibiotics are often administered, usually including beta-lactams with anaerobic coverage as the first line of defense, however, culture and sensitivity testing is important as one study found that nearly half of the 96 dogs tested (46%) required an antibiotic change based on susceptibility testing (77). Notably, fluoroquinolone-resistant *S. pseudintermedius* was isolated from a recurrent subcutaneous abscess in a cat that contained genes for leukocidins (lukS and lukF), exfoliative toxin (seit) and biofilm regulation (icaA and icaD). The isolate was also found to be resistant against macrolide, lincosamide and tetracycline classes (80).

## 4 Conventional antibiotic use in veterinary dermatology

Antibiotics have been a vital component within veterinary medicine and for the treatment of bacterial skin infections in companion animals. Among the most frequently prescribed systemic antibiotics are beta-lactams, including penicillins and cephalosporins (notably cephalexin and cefpodoxime), fluoroquinolones (such as enrofloxacin and marbofloxacin), and lincosamides like clindamycin (81). Topically administered antimicrobials are also widely used, with fusidic acid, mupirocin, and antiseptics like chlorhexidine and benzoyl peroxide commonly used in topical therapy (82). In Australia, it was found that approximately one-third of veterinary consultations involved an antibiotic prescription, with a marked preference for broad-spectrum agents (83). A French study analyzing veterinary prescription records from 2014 to 2020 similarly revealed high usage of critically important antimicrobials such as third-generation cephalosporins and fluoroquinolones (84). The consistent usage of antibiotics has led to a selective pressure on both pathogenic and commensal bacteria, resulting in the prevalence of antimicrobial-resistance and the spread of resistant genes (Table 1) (14). This can lead to increased recurrence rates and therapeutic failures (84, 85). One striking example is the rising prevalence of methicillin-resistant *Staphylococcus pseudintermedius* (MRSP), a multidrug-resistant organism that has been increasingly isolated in canine skin infections globally (4, 53, 86). *Staphylococcal* species exhibit resistance to multiple drug classes, including beta-lactams, macrolides, tetracyclines, and fluoroquinolones (87, 88). These resistance mechanisms are gene-encoded and include the production of beta-lactamase enzymes (e.g., *blaZ*), efflux pumps (e.g., *Nor*A), and alterations in antibiotic target sites (e.g., PBP2a conferred by *mec*A) (50, 51, 89). These adaptations not only limit treatment options but may also facilitate transmission of resistant organisms within veterinary hospitals and between animals and humans, making the zoonotic potential of AMR pathogens a critical concern (86). Close contact between humans and companion animals allows for the bidirectional exchange of resistant bacteria, as demonstrated in several studies involving MRSP and extended-spectrum beta-lactamase (ESBL)-producing *Enterobacteriaceae* (90–92). In contrast, the use of evidence-based prescribing involving laboratory diagnostics to guide antimicrobial selection is associated with a more targeted therapy for the animal, reduced resistance development, and improved clinical outcomes. This approach is underutilized due to factors such as cost, time constraints, patient demand and limited access to diagnostic facilities (13).

**Table 1:** Common resistance genes and mechanisms in bacterial pathogens of canine and feline skin infections.

Resistance gene/Element	Mechanism	Drug class affected	Example organism(s)
mecA/mecC (SCCmec)	Altered target: PBP2a with low β-lactam affinity	β-lactams (methicillin)	*S. pseudintermedius, S. aureus*
blaZ	β-lactamase (penicillin hydrolysis)	Penicillins	*Staphylococcus* spp.
erm (e.g., ermB/C)	rRNA methylation of the ribosomal target	Macrolides, lincosamides, streptogramin B	*Staphylococcus* spp.
tet(K)/tet(M)	Efflux pump/ribosomal protection	Tetracyclines	*Staphylococcus* spp.
norA	Efflux pump	Fluoroquinolones (and biocides)	*S. aureus*
AmpC; mexAB–oprM, mexXY; oprD loss	Chromosomal β-lactamase; efflux up-regulation; porin loss	β-lactams, aminoglycosides, fluoroquinolones	*P. aeruginosa*

## 5 Propolis as a natural antimicrobial agent

The *in vitro* antibacterial activity of propolis and its individual components is well documented against numerous Gram-positive and Gram-negative bacteria (30). However, studies have consistently shown that propolis exhibits more potent activity against Gram-positive bacteria, such as *S. aureus*, *Streptococcus* spp.*, Listeria monocytogenes* (*L. monocytogenes*), *Bacillus subtilis* (*B. subtilis*), and *Enterococcus faecalis* (*E. faecalis*) (19, 93). This heightened susceptibility is largely attributed to the absence of an outer membrane in Gram-positive bacteria, which allows the bioactive compounds in propolis to more readily penetrate and disrupt bacterial cell walls (94). Although Gram-negative bacteria generally exhibit greater resistance due to their outer membranes and efflux mechanisms, certain propolis extracts have demonstrated modest inhibitory activity against these organisms (95–97). For instance, ethanol extracted Egyptian propolis has shown antibacterial activity against *P. aeruginosa*, *K. pneumoniae* and *E. coli* with minimum inhibitory concentration (MIC) values of 14.66, 15.63 and 31.25 mg/mL, respectively (98). Red Brazilian propolis fractions demonstrated activity against *E. coli* and *P. aeruginosa* with MIC values of 454 and 227 μg/mL, respectively (99). Although these concentrations are considerably higher than those effective against Gram-positive pathogens, they indicate that propolis retains some activity against Gram-negative bacteria.

The antimicrobial activity of propolis is attributed to its complex chemical composition, particularly, its flavonoids, phenolic acids, esters and terpenoids which can act through multiple mechanisms simultaneously or synergistically to disrupt bacterial viability and virulence (Figure 3) (98). These mechanisms include disruption of the bacterial cell membrane integrity, interfering with metabolic processes and suppression of efflux pumps (100). The flavonoid compound pinocembrin, found in propolis, has been shown to inhibit bacterial pathogens, including *Aeromonas hydrophila*, through increasing cell membrane permeability and affecting protein and DNA metabolism (35). Caffeic acid esters from Egyptian propolis displayed strong bactericidal activity and inhibited bacterial growth by disrupting membrane permeability and through oxidative stress mechanisms (98). Propolis rich in cinnamic acid and its derivates similarly damage cell membranes while also inhibiting ATPase activity, cell division and biofilm formation (101). Additionally, several studies have demonstrated that flavonoid-rich propolis extracts inhibit bacterial DNA gyrase and topoisomerase IV, enzymes critical for bacterial DNA replication and transcription. For example, quercetin was found to bind to the subunit of *E. coli* DNA gyrase to inhibit the ATPase activity (33). By disrupting nucleic acid synthesis, propolis impairs the ability of bacteria to proliferate and spread infection. Another key antimicrobial property is the potential for propolis to suppress the function of bacterial efflux pumps. These pumps are often upregulated in multidrug-resistant organisms to expel antimicrobial agents from within the bacterial cell (32). Brown propolis fractions from Northern Ceará, Brazil were found to have potentiating effects when used in combination with Norfloxacin which was attributed to efflux pump inhibition (102). A study by Al-Sallami et al. (103) demonstrated that propolis had a significant synergistic effect in combination with ciprofloxacin, erythromycin and gentamicin with an ethidium bromide accumulation assay showing inhibitory activity against *S. aureus* pump system. Inhibition of these systems would enhance intracellular drug accumulation in the cell, opening up the potential efficacy for the use of both propolis and conventional antibiotics in combination (104). This multifactorial mode of action is a key distinguishing feature of propolis compared with conventional antibiotics, which typically act on a single molecular target. By simultaneously disrupting membrane integrity, nucleic acid synthesis, efflux activity and biofilm formation, propolis may reduce the probability of bacteria developing resistance through a single gene mutation (105). Furthermore, the demonstrated synergy between propolis and conventional antibiotics, such as enhancing intracellular drug accumulation through efflux pump inhibition, suggests that propolis may have a role not only as a standalone antimicrobial but also as an adjunct that restores or enhances the efficacy of existing antibiotics against resistant organisms. This is particularly relevant to veterinary dermatology, where multidrug resistant pathogens such as MRSP and *P. aeruginosa* present significant treatment challenges (4, 91).

**Figure 3:**
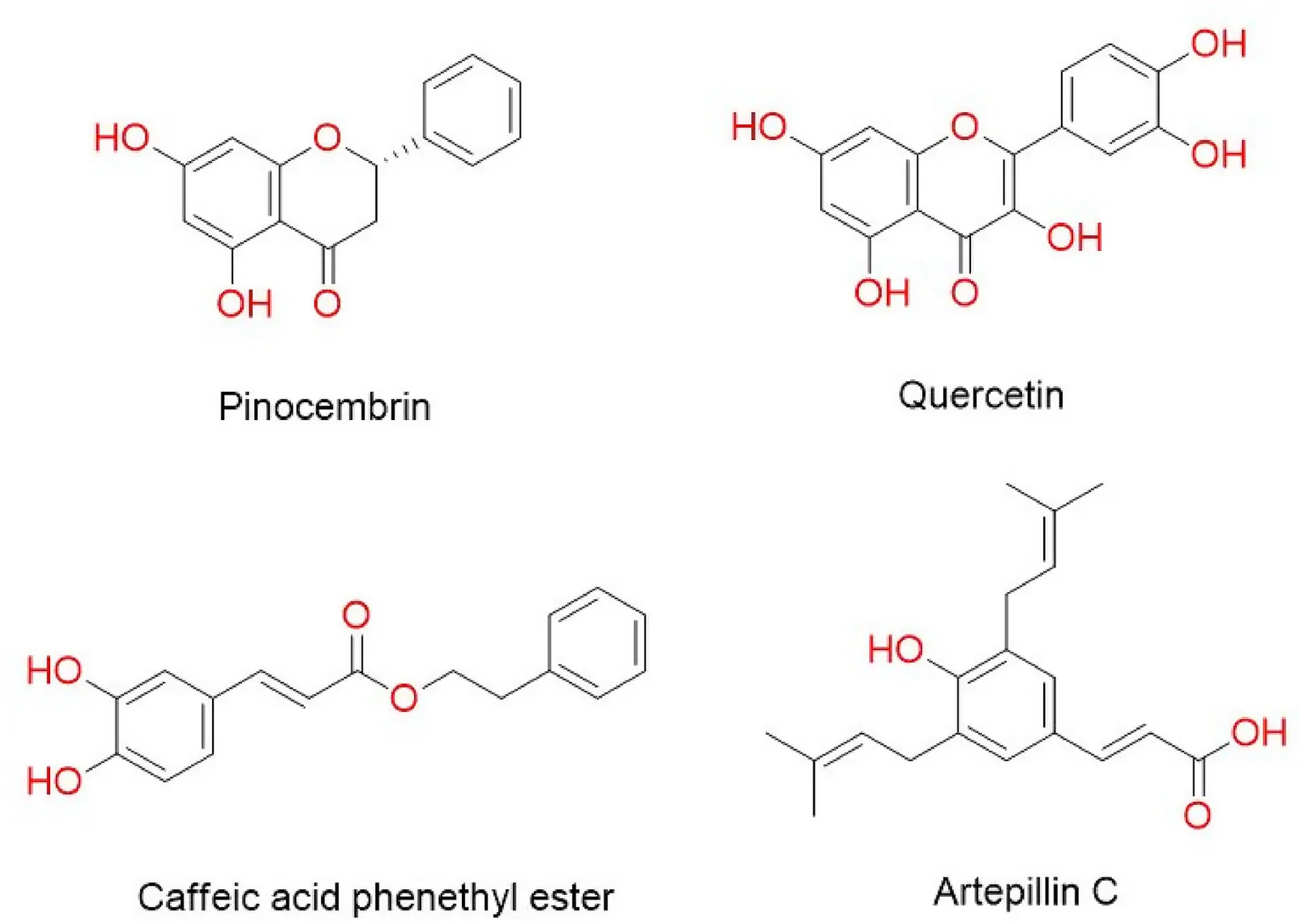
Chemical structures of some known antibacterial compounds identified from propolis (generated in ChemSketch).

## 6 Evidence of propolis use in veterinary dermatology

Several studies have demonstrated that propolis exhibits antimicrobial activity against several pathogens implicated in veterinary dermatology. The antibacterial activity of propolis against companion animal skin pathogens has been evaluated across multiple studies using propolis from diverse geographic origins. Romanian propolis demonstrated potent activity against *S. aureus* strains isolated from dogs with superficial pyoderma, with MIC values ranging from 6–10 μg/mL, including methicillin-resistant strains (19). Brazilian propolis has similarly exhibited antibacterial activity, with Green propolis inhibiting *S. pseudintermedius* isolated from canine pyoderma with an MIC of 156 μg/mL and minimum bactericidal concentration (MBC) of 312 μg/mL (36). Another study examined Brazilian propolis antibacterial activity against coagulase-positive *Staphylococci* isolated from canine otitis externa and found the MBC required to kill 90% (MBC90) was 21,000 μg/mL (106). Propolis from the Baltic region was extracted using deep eutectic solvents (DES) and found broad-spectrum activity against *S. aureus*, *S. agalactiae*, *B. cereus*, *E. faecalis*, *E. coli* and *P. aeruginosa* isolated from small animals with acute superficial or deep pyoderma, wounds infections, abscesses and otitis with MIC values of 100, 200, 200, 500, 1,000 and 1,000 μg/mL, respectively (104). Notably, the DES-based extract exhibited stronger antimicrobial activity compared to the corresponding ethanolic extract spanning from 500–5,000 μg/mL, highlighting that the extraction method can significantly influence antimicrobial potency. Argentinean propolis extracts were effective against several *Staphylococcus* species isolated from canine otitis with MIC values with MIC values ranging from 10–80 μg/mL (38). Furthermore, another study investigated Argentine propolis as an ear drop formulation against canine externa and found antimicrobial activity against the isolated microorganisms *S. aureus*, *E. faecalis*, *P. aeruginosa*, and *P. mirabilis*. and had a 71% recovery rate from the canine patients (107). Portuguese propolis demonstrated activity against several *Staphylococcus* species, *E. coli* and *Klebsiella oxytoca* (*K. oxytoca*) isolated from skin and ear infections from both cats and dogs. The MIC values ranged from 500–1,000 μg/mL for the *Staphylococcus* species and 8,000 μg/mL for both *E. coli* and *K. oxytoca* (108). Additionally, this study found no adverse effects of propolis in a *Galleria mellonella* toxicity model.

Propolis has also demonstrated activity against fungal veterinary dermatologic pathogens including dermatophytes and the yeast *Malassezia* spp. (Table 1). A 2014 study presented three clinical cases of canine dermatophytosis that resolved following topical propolis treatment (109). Radial growth inhibition assays on the isolated cultures showed that two strains of *Microsporum canis* were inhibited at all concentrations tested, while *M. gypseum* was inhibited at 2, 4 and 8 μg/mL, with an MIC of 500 μg/mL for all three strains (109). These *in vitro* findings provided preliminary evidence of antifungal activity against the dermatophytes isolated from the affected dogs and were followed by clinical observations in three individual canine cases (*n* = 1 per case) treated with topical propolis (109). The first case involved *M. gypseum*, while the second and third involved *M. canis*. However, the clinical cases did not include concurrent untreated, placebo, or conventional antifungal control groups, and the authors did not report randomization or blinding of the investigators, clinicians, owners, or outcome assessors. Clinical outcomes were assessed based on resolution of skin lesions, reduction in pruritus, and hair regrowth, while microbiological outcomes were monitored through weekly hair sampling, direct KOH examination, and fungal culture. Cases 1 and 2 were treated for approximately 3 weeks, whereas Case 3 required 8 weeks of treatment, with assessments conducted every 7 days (109). Although all three cases reportedly showed clinical improvement and negative fungal cultures, the clinical findings should be interpreted cautiously because of the very small sample size, absence of a clinical control group, lack of reported blinding or randomization, and variable treatment and follow-up durations. Thus, while the *in vitro* results and subsequent clinical observations are consistent with potential antifungal activity of topical propolis against *Microsporum* spp., they provide preliminary evidence rather than robust evidence of clinical efficacy. Supporting this, Turkish propolis was assessed against dermatophytosis causing microorganisms isolated from cats. At concentrations ranging from 6.25 to 1,000 mg/mL, the propolis achieved 100% inhibition of mycelial growth (39). Galinari et al. (110) tested a novel propolis-based formulation against several dermatophyte species including *M. canis*, *Nannizzia gypsea*, *T. mentagrophytes*, and *T. rubrum*. They incorporated propolis into a bioadhesive thermoresponsive gel containing 16% propolis to improve its application in a topical therapy. Both the propolis gel and propolis extract alone significantly reduced the viability of all tested dermatophyte strains *in vitro*. In addition, the propolis formulation was found to interact with and disrupt dermatophyte biofilms which can contribute to chronic or resistant infections (110). Another study used a 10% propolis ointment to treat dermatophytosis in rabbits and compared the effectivity against the antibiotic Miconazole 2% and found that the propolis had a greater reduction in inflammation and was able to heal the lesions with the same recovery time as the antibiotic (110). Antifungal activity against *Malassezia* spp. has also been reported with one study reporting Mexican stingless bee propolis with an MIC of 7.11 mg/mL and Minimum Fungicidal Concentration (MFC) of 21.33 mg/mL (37) and another study reporting an MFC90 of 5.3 mg/mL for Brazilian propolis (106).

These *in vitro* results consistently show that propolis possesses meaningful antibacterial and antifungal activities against the key pathogens of veterinary skin disease. The broad efficacy of propolis against Gram-positive bacteria, yeasts, and dermatophyte fungi underscores its potential as a therapeutic option in veterinary dermatology, warranting further investigation in clinical settings. Furthermore, recognizing the need for clinically applicable delivery systems, several studies have investigated propolis-based formulations for topical veterinary use. Galinari et al. (110) incorporated propolis into a 16% bioadhesive thermoresponsive gel and found that both the gel and propolis extract alone reduced the viability against all tested dermatophyte species as well as disrupted fungal biofilms. Svetikienė et al. (20) developed hydrogels, oleogels and bigels using DES-extracted propolis incorporated into poloxamer 407-based formulations at 13%, 15 and 18% concentrations. Of these, the hydrogel formulation released the highest concentration of phenolic compounds and demonstrated the strongest antibacterial activity against bacterial pathogens isolated from small animals with pyoderma, wound infections and abscesses (20). Sánchez et al. (109) tested a combination of topical propolis soap baths as well as propolis ointment with all lesions resolved. These formulations studies represent an important step toward translating the *in vitro* findings into practical topical treatments, although more clinical evaluation in companion animals needs to be conducted. Currently, there are several propolis-based veterinary products on the market targeted primarily on dogs and cats (Supplementary Table S1). These include topical preparations such as ear drops, skin balms, wound sprays and healing powders as well as oral supplements for immune support.

While these studies collectively support the antimicrobial potential of propolis in veterinary dermatology, there are several limiting factors that need to be considered. The majority of the available evidence is derived from *in vitro* studies, which although valuable, does not account for the complexity of a biological environment of a living host. Antimicrobial values reported across the included studies vary considerably. The *in vivo* and clinical evidence studies is limited both in quantity and scope. Of the three studies included, two addressed dermatophytosis (107, 109) and one addressed otitis (111). There is a notable lack of *in vivo* studies investigating propolis for the treatment of pyoderma, abscesses and bite wounds. Additionally, the variability in propolis composition across the studies needs to be considered as propolis is a heterogeneous material (29) and were sourced from regions including Brazil, Argentina, Romania, the Baltic states, Portugal, Turkey and Iraq. Extraction methods also varied considerably, ranging from standard ethanolic extraction to deep eutectic solvents and nano-formulations, each yielding different concentrations of bioactive compounds and, consequently, different antimicrobial potencies. This heterogeneity makes direct comparison of results across studies problematic.

## 7 Propolis as an alternative or adjunct to conventional antimicrobials

Conventional antimicrobials used in veterinary dermatology are associated with a range of limitations beyond resistance development. Systemic antibiotics like amoxicillin-clavulanate, clindamycin or enrofloxacin can cause side effects such as gastrointestinal upset and can disturb the normal gut flora (112). Topical antibiotics and antiseptics are also implicated with adverse effects which may cause irritation or allergic reactions, for example, neomycin and bacitracin are known to cause contact allergic reactions (113, 114), and prolonged chlorhexidine use can dry out the skin (115). Systemic antifungal used or dermatophytosis, particularly ketoconazole and itraconazole, carry a risk of hepatoxicity and require extended treatment courses of 6–8 weeks (116). Propolis as an alternative treatment option to conventional antibiotics can offer several advantages. This includes the multiple modes of action which the complex mixture of propolis can enact at once, undermining bacteria’s ability to develop resistance. Additionally, propolis has demonstrated efficacy against resistant pathogens, for example, through the destruction of efflux pumps (102). Conventional antibiotics typically act on a single cellular pathway, for instance, β-lactam antibiotics block cell wall synthesis, and fluoroquinolones inhibit DNA gyrase or topoisomerase IV (30). Another advantage that propolis offers over conventional antibiotics is propolis’ ability to exert ancillary immunomodulatory effects. Some propolis components, e.g., artepillin C, a phenolic compound, can modulate host inflammatory pathways by inhibiting NF-κB activation and suppressing pro-inflammatory mediators like prostaglandin E2 and nitric oxide (117). This anti-inflammatory action may contribute to faster resolution of dermatologic lesions which is something standard antibiotics do not provide. Furthermore, propolis, as a therapeutic agent, appears to have a favorable safety profile. Rodrigues et al. (108) assessed the safety of propolis using a *Galleria mellonella* model and found no adverse effects although the predictive value of this model for mammalian toxicity demonstrates inherently limited. Aldana-Mejia et al. (118) tested Brazilian red propolis via genotoxicity assays in mice and found no genotoxic effects. At a target animal level, a study from Cho (119) assessed liquid and powder administration of propolis in beagle dogs over an 8-week period and found no significant clinical signs or changes. Comparable controlled tolerability data, however, is lacking. *In vitro* toxicology assessments on canine skin cell lines have shown that propolis concentrations effective against bacteria have minimal harmful effect on keratinocytes and fibroblasts (120). It is important to note that propolis is not entirely devoid of side effects with the potential for allergic reactions. Propolis contains allergenic components such as certain caffeate esters and cases of contact dermatitis have been documented, although primarily in humans and more specifically, from beekeepers who have been repeatedly exposed to raw propolis rather than the pure propolis extract (121).

## 8 Future directions

The findings of this review highlight several priority areas for future research. A significant challenge in developing propolis-based therapeutic products is standardization and quality control (29). Variations in the chemical composition and biological activity of different propolis types may lead to inconsistent therapeutic outcomes, thereby hindering reproducibility and complicating regulatory approval processes (122, 123). Therefore, to fully realize the potential of propolis as a largely untapped natural resource for combating antimicrobial resistance and developing sustainable therapies, it is essential to isolate and characterize active compounds to identify chemical markers associated with biological activity as well as to establish standardized extraction protocols that ensure consistent concentrations of key bioactive constituents (124). Such efforts will facilitate product standardization, reproducibility, and regulatory acceptance while enhancing our understanding of the synergistic effects between propolis and conventional antibiotics (125). Additionally, research is needed for formulation development to optimize the delivery systems of propolis in dermatologic applications (126). Despite promising *in vitro* antimicrobial activity, the successful translation of propolis into clinically effective therapies remains challenging. Key considerations include the limited capacity of MIC/MBC data to predict *in vivo* efficacy, the barrier function of the stratum corneum, and the effects of wound exudate and tissue pH on propolis activity. Furthermore, formulation development must address critical parameters such as optimal pH, viscosity, and stability throughout storage and use to maximize therapeutic effectiveness and ensure consistent product quality (20). Future research should continue to explore novel carriers and formulation techniques including hydrogels, creams, soaps, ointments, nanoparticles, or slow-release dressings that maximize bioavailability and therapeutic effect on the skin (127). Lastly, a vital research gap is the lack of rigorous clinical trials to validate propolis’ efficacy and safety in companion animals highlighting the need for well-designed randomized controlled trials with appropriate outcome measures and adequate sample size considerations. Although propolis is generally considered low in toxicity, further studies are required to assess its allergenicity and the safety of long-term administration in target species. Future clinical investigations should address practical considerations relevant to veterinary application, including treatment cost, owner compliance, frequency of administration, and whether propolis-based therapies are best positioned as first-line treatments or reserved for cases involving antimicrobial resistance. Such evidence will be critical for informing clinical decision-making and supporting the translation of propolis into routine veterinary practice. Additionally, research into the treatment of skin infections in companion animals has largely centered around propolis derived from the European honeybee (*Apis mellifera*). There is limited exploration of propolis from Asian honeybees and stingless bees, despite evidence of their strong antimicrobial activity against human pathogens (37, 128–132) highlighting a promising opportunity to develop effective treatments from these propolis types. Given this variability, defining minimum standardization criteria will be a prerequisite for any future veterinary clinical use of propolis. At a minimum, propolis-based preparations intended for therapeutic evaluation should be accompanied by (i) declaration of botanical and geographical origin and of the producing bee species; (ii) a validated chemical fingerprint obtained by a reproducible analytical method such as HPLC, LC–MS or HPTLC; (iii) quantification of defined marker compounds together with total phenolic and flavonoid contents, with candidate markers such as caffeic acid phenethyl ester, artepillin C, galangin and pinocembrin selected according to propolis type; (iv) demonstrated batch-to-batch consistency against pre-specified acceptance ranges for these markers; and (v) a fully reported, standardized extraction protocol specifying the solvent, solvent-to-propolis ratio, temperature and duration (29, 133). Establishing such criteria would improve reproducibility across studies, permit meaningful comparison of antimicrobial potencies, and support a regulatory approval pathway for propolis-based veterinary products.

## 9 Conclusion

Companion animal skin infections, particularly pyoderma and otitis, are significant challenges in veterinary medicine. This often leads to over-prescription practices contributing to the development of antimicrobial-resistant pathogens. This review identified 18 studies evaluating propolis for the treatment of dermatologic infections in companion animals with the majority of studies conducted in dogs. The available evidence indicates that propolis demonstrates antimicrobial activity against key veterinary skin pathogens including *Staphylococcus* spp., *P. aeruginosa*, *Malassezia* spp. and dermatophytes. Its complex chemical composition, comprising flavonoids, phenolic acids and terpenoids, enables multiple simultaneous mechanisms of antibacterial action, which may reduce the likelihood of resistance development compared with single-target conventional antibiotics, though this has not yet been directly demonstrated through longitudinal resistance studies. Propolis also offers ancillary benefits not provided by conventional antimicrobials, including anti-inflammatory and immunomodulatory activity and a generally favorable safety profile although evidence supporting the clinical significance of these effects in companion animals remains limited. Therefore, more research is needed to determine its full therapeutic potential. Priorities include conducting controlled clinical trials, developing standardization extraction protocols, identification of key bioactive markers and refining delivery systems. This research potentially offers significant benefits in veterinary dermatology and offers broader contributions for antimicrobial stewardship efforts aligned with the One Health initiative.

## Footnotes

1
https://new.rayyan.ai/
