# Supplementary material for: Propolis as an alternative treatment for companion animal skin infections: addressing antimicrobial resistance
Source: Front Vet Sci. 2026 Oct 22;13:1880076. doi: 10.3389/fvets.2026.1880076 (PMC13735108; doi:10.3389/fvets.2026.1880076)
Supplement: Supplementary file 1 [file Table_1.DOCX]

**Supplementary Table S1.** Commercially available propolis-based veterinary products.

| **Product name** | **Formulation** | **Route** | **Intended use** | **Active ingredients** | **Manufacturer (country)** |
| --- | --- | --- | --- | --- | --- |
| Mānuka Vet® Wound Spray | Topical spray | Topical | Antimicrobial wound care for cuts, abrasions, paw injuries and hot spots | Medical-grade mānuka honey (20%), propolis, aloe vera, lemongrass oil | Manuka Vet (New Zealand) |
| HoneyPro® Honey Wound Wax | Topical salve/ointment | Topical | Wound healing for cuts and sores in companion animals and livestock | Mānuka honey, propolis, calendula extract, vitamin A (wax base) | HoneyPro Vet (Australia) |
| Propolia® Paw & Skin Balm | Topical balm | Topical | Skin and paw pad repair; management of dry, cracked skin and minor wounds | Propolis, shea butter, honey | Apimab Laboratoires – Propolia (France) |
| Propolia® Propolis Skin Care Powder | Fine powder | Topical | Wound drying and healing support; promotion of protective scab formation | Propolis, horsetail extract | Apimab Laboratoires – Propolia (France) |
| Excellent Pets® Propolis Spray | Topical spray | Topical | Skin care for minor irritations, scratches and dry patches | Propolis extract | Excellent Pets (Netherlands) |
| Excellent Pets® Propolis & Honey Ointment | Ointment | Topical | Support of skin restorative capacity and general skin care | Propolis, honey | Hofman Animal Care (Netherlands) |
| Bleu & Marine® VET Propolis Repair Gel | Topical gel/cream | Topical | Wound healing and skin care for companion animals and horses | Propolis, arnica gel, aloe vera gel | Bleu et Marine Bretania (Spain) |
| Propolia® Ear Care Drops | Topical ear drops | Topical (aural) | Ear hygiene; soothing of irritation and earwax removal | Propolis, calendula extract, tea tree oil | Apimab Laboratoires – Propolia (France) |
| Bellfor® Immun Powder | Oral powder | Oral | Immune support for adult, senior or stressed dogs | Insect protein, propolis (~4%), nettle seed, horsetail, rosehip | Bellfor (Germany) |
| ReaVET® Propolis Powder | Oral powder | Oral | Immune system and skin health support | 100% ground bee propolis (food-grade), maltodextrin carrier | ReaVET (Switzerland/Germany) |
| AniForte® Propolis Powder | Oral powder | Oral | Immune system, defence and skin vitality support | Propolis powder, maltodextrin | AniForte (UK) |
| Pure Herbs® Bee Propolis Tincture | Oral liquid tincture | Oral | Nutraceutical for immune support; antioxidant supplement | Propolis extract (glycerine-based) | Pure Herbs Ltd. (USA) |
| Mueller Honeybee® Propolis Tartar Paste | Oral paste | Oral (dental) | Dental care; reduction of tartar and support of gum health | Propolis, organic coconut oil, diatomaceous earth, myrrh, grapefruit seed extract, neem | Mueller Honey Bee (USA) |

Product information sourced from manufacturer websites, accessed [03-03-2026]. Inclusion in this table does not constitute endorsement of any product. None of the listed products have published peer-reviewed clinical trial data supporting their claimed efficacy for the treatment of dermatological infections in companion animals. Products are grouped by administration route and intended use. This table is intended to illustrate the current commercial landscape and is not used to support claims of antimicrobial efficacy within this review.
